# Advancing data-driven health research from the *All of Us* data training and engagement program

**DOI:** 10.5195/jmla.2026.2324

**Published:** 2026-07-01

**Authors:** Kristi Sadowski, DeBran Tarver, Jennifer Burnette, Allissa Dillman, Regina Renfro, LaFrancis Gibson, Kelli Bursey, Laura Bartlett, Nadine Bonds-Bishop, Lisa Connor, Efraín Flores-Rivera, Sarah Joseph, Yuqi He, Margaret Henderson, Savannah L. Kelley, Maletta Payne, Brett Porter, Kimberly Prosper, Melissa Rethlefsen, Deborah J. Rhue, Dede Rios, Anna Simonson, Jocelyn Swick-Jemison, Linda Todd, Shelby Watson, Semhar Yohannes

**Affiliations:** 1 k.m.sadowski@gmail.com, Project Coordinator, Public Health and Healthcare, Oak Ridge Associated Universities (ORAU), Oak Ridge, TN; 2 debran.tarver@gmail.com, Program Evaluator, Public Health and Healthcare, Oak Ridge Associated Universities (ORAU), Oak Ridge, TN; 3 Jennifer.Burnette@orau.org, Project Director, Public Health and Healthcare, Oak Ridge Associated Universities (ORAU), Oak Ridge, TN; 4 adillman@biodatasage.com, Principal Scientist, BioData Sage LLC, Bethesda, MD; 5 Regina.Renfro@orau.org, Communication Specialist, Public Health and Healthcare, Oak Ridge Associated Universities (ORAU), Oak Ridge, TN; 6 Lafrancis.Gibson@orau.org, Program Manager, Public Health and Healthcare, Oak Ridge Associated Universities (ORAU), Oak Ridge, TN; 7 Kelli.Bursey@orau.org, Program Evaluator, Public Health and Healthcare, Oak Ridge Associated Universities (ORAU), Oak Ridge, TN; 8 bartlettl@mail.nlm.nih.gov, Technical Information Specialist, National Library of Medicine, National Institutes of Health, Bethesda, MD; 9 nbonds@twu.edu, Library Manager / Health Sciences Librarian, Texas Woman's University Institute of Health Sciences-Houston, Houston, TX; 10 lconnor@shsu.edu, Assistant Professor, Research & Instruction, Sam Houston State University, Huntsville, TX; 11 efrain.flores@upr.edu, Library Director, Conrado F. Asenjo Library, University of Puerto Rico, Medical Sciences Campus, San Juan, PR; 12 josepsar@gvsu.edu, Liaison Librarian for Biomedical Sciences, Exercise Science, Health Informatics & Bioinformatics, Movement Science, Public Health, and Statistics & Biostatistics, University Libraries, Grand Valley State University, Allendale, MI; 13 yuqi.he@sjsu.edu, Engineering and Data Services Librarian, Dr. Martin Luther King Jr. Library, San José State University, San Jose, CA; 14 margaret.henderson@sdsu.edu, Health Sciences and Research Data Services Librarian and Head of Research, Instruction, and Outreach Services, University Library, San Diego State University, San Diego, CA; 15 slkelly@olemiss.edu, Associate Head and Associate Professor of Scholar Support and Data Services, University of Mississippi, University, MS; 16 maletta.payne@sus.edu, Associate Professor and Head, Technology and Information Services Librarian, Southern University and A&M College, Baton Rouge, LA; 17 bxp040@shsu.edu, Assistant Professor, Newton Gresham Library, Sam Houston State University, Huntsville, TX; 18 kprosper@howard.edu, Howard University, Washington, DC; 19 mlrethlefsen@gmail.com, Dean and Professor, Health Sciences Library and Informatics Center, University of New Mexico Health Sciences Center, Albuquerque, NM; 20 deborahrhue@gmail.com, Clinical Services Librarian, Retired, Health Sciences Library and Informatics Center, University of New Mexico Health Sciences Center, Albuquerque, NM; 21 dmrios1@uiwtx.edu, Director of Public Services and Community Health, Nursing, and Mexico Campuses Liaison Librarian, UIW Libraries, University of the Incarnate Word, San Antonio, TX; 22 anna.simonson@usd.edu, Associate Dean for Teaching, Learning, and Research Services and Associate Librarian, University Libraries, University of South Dakota, Vermillion, SD; 23 jswick@buffalo.edu, Data Services Librarian (Health Sciences), University Libraries, University at Buffalo, Buffalo, NY; 24 Assistant Professor, ltodd@marymount.edu Instruction and Reference Librarian and Reference Coordinator, Emerson G. Reinsch Library, Marymount University, Arlington, VA; 25 sahilton@olemiss.edu, Assistant Professor of Scholar Support and Data Services, University of Mississippi, University, MS; 26 semhar@umbc.edu, Health Sciences and Human Services Librarian, Albin O. Kuhn Library & Gallery, University of Maryland, Baltimore County, Baltimore, MD

**Keywords:** Data training, Biomedical Informatics, Research Capacity Building, Academic Libraries, Data Literacy, NIH, *All Of Us* Research Program, Data Access, Data-Driven Research, Biomedical Data, Public Health Data

## Abstract

**Background:**

Fulfilling a recognized need in data skills training for academic librarians, the National Library of Medicine and the National Institutes of Health *All of Us* Research Program collaborated to enhance academic library workers& skills in biomedical and public health data, as well as their library's research capacity, through the awareness and use of the *All of Us* Researcher Workbench.

**Case Presentation:**

The *All of Us* Data Training and Engagement Program for Academic Libraries (ALP) blended professional development training, hands-on learning, and peer-to-peer networking that focused on increasing knowledge of the *All of Us* Researcher Workbench. Activities were designed to build institution-wide, interdisciplinary awareness and interest in using the *All of Us* Researcher Workbench. A series of ongoing activities ensured sustained skill-building and collaboration, and included training for R, a program language used for statistical computing and data visualization. Program activities were intentionally designed to help grow institutional research capacity, enhance skills in biomedical and public health data, and promote meaningful use of the Researcher Workbench to campus communities.

**Conclusion:**

The ALP helped participants overcome barriers to data access and improve research infrastructure and successfully empowered 115 library professionals to leverage the *All of Us* Researcher Workbench for meaningful biomedical and public health research. Measured outcomes validate the success of the program and demonstrate how the ALP has positioned participating institutions for long-term success in biomedical and public health research. Institutions can build upon the foundation established through this case report to advance equitable, data-driven health research across academic landscapes.

## BACKGROUND

The *All of Us* Research Program is a historic effort to collect and study data from one million or more people living in the United States, and the *All of Us* Research Hub stores health data from a diverse group of participants from across the United States. The *All of Us* Researcher Workbench is a cloud-based platform with tools to support data analysis and collaboration and combines biological factors and social determinants on a large inclusive scale that follows participants as they move, age, and grow. The data is ideal for researchers from a wide range of disciplines, making it especially valuable to librarians as a campus data resource (“Data Sources – *All of Us* Research Hub”).

Librarians have been framed as essential human support to complement advances in technical and computing infrastructure [1], and are strong partners in championing researchers and campus partners in research data support. Federer et all describes librarians as “data people with interpersonal skill sets” who can meet the data science-related needs of researchers [2]. Despite being positioned for assisting researchers, 77% reported they had not received training in data literacy [3]. To bridge this gap, the National Library of Medicine (NLM) and the National Institutes of Health (NIH) *All of Us* Research Program collaborated to create the *All of Us* Data Training and Engagement Program for Academic Libraries (ALP), in 2021.

Funded for years 2023 and 2024 the ALP, managed by Oak Ridge Associated Universities (ORAU), aimed to provide an opportunity for academic libraries and library workers to be part of the *All of Us* historic and collaborative effort to improve the health and health care of individuals living in the United States.

## CASE PRESENTATION

### Academic Libraries Program Overview

The ALP worked to enhance library workers’ skills in biomedical and public health data and improve the research capacity of the campus libraries through engagement in a community-learning cohort. Program creation was informed by a comprehensive mixed-methods needs assessment, identifying five overarching program goals to assess campus opportunities to encourage use of the *All of Us* dataset, including:

1. Identify facilitators, barriers, and opportunities.
2. Provide training opportunities for library workers to build skills and capacity.
3. Position the library as a place for the campus community to gain *All of Us* data knowledge, skills, and abilities.
4. Develop a resource to raise awareness and communicate the availability of the Researcher Workbench.
5. Provide a series of data engagement activities for library workers to build skills to provide data engagement.

A total of 25 institutions participated in the program, with 15 being part of a pilot employing perpetual evaluation to refine and improve. The 15 institutions in the pilot had slightly different requirements as their feedback refined the program for 10 institutions in Cohort 2. The Pilot Cohort focused on Minority Serving Institutions (MSIs), and Cohort 2 recruited institutions with a historic and current commitment to, or track record of, educating students that are underrepresented in biomedical careers. See Appendix A for institutions by cohort.

### Program Activities

The ALP designed activities focused on increasing participants’ knowledge of the *All of Us* Researcher Workbench and included hands-on learning and funding to support data-related infrastructure needs and research capacity building within campus libraries. Activities were developed to showcase methods for engagement with the Researcher Workbench and included data skills training and campus-wide promotions to help build institution-wide, interdisciplinary awareness and interest in the Researcher Workbench. An additional series of ongoing activities was planned to ensure sustained skill-building and collaboration. All participants were expected to complete short evaluation surveys after each training session and at strategic points throughout the ALP. Appendix B outlines the key components and milestones of ALP activities.

### Program Assistance and Requirements

#### Facilitation

Federer et al. (2020) state that library leaders are essential for supporting data science/open science services. Library leaders must make critical staffing decisions for meeting the institution’s needs, advocate for library services, and raise awareness of data science/open science services at high levels in the institution [4]. ALP participants engaged leadership by informing them of participation, progress, and key decision-making. Program administrators were required to complete mid-cycle and final reports tracking the impact of the ALP program.

A participant dashboard using a Microsoft Teams Channel fostered peer-to-peer networking outside of trainings and cohort meetings. This platform also acted as a repository for training materials and templates.

#### Training and Cohort Meetings

Online trainings were offered on topics including data engagement activity examples and usage of the Researcher Workbench for researchers and with campus communities. Descriptions for trainings with learning objectives can be found in Appendix C.

Cohort meetings offered presentations and facilitated discussions on potential applications for the capacity building award, developing a resource guide, assessing researcher needs, managing cloud computing and billing, coordinating cohort activities, working with faculty, and practicing self-directed R coding practices.

#### Capacity Building Plans & Awards

Program administrators developed a Capacity Building Plan based on a $40,000 per institution award that would build or enhance research capacity using the Researcher Workbench. Plans were intended to be flexible by allowing each administrator to identify the needs of their specific institution.

### Program Evaluation

Evaluation methods employed a logic model-driven, mixed-methods design to assess program impact and outcomes. The evaluation included a formative component aimed at refining program design.

Six mixed-methods approaches were implemented for evaluation. Methods included qualitative onboarding questions conducted via group sessions and surveys, infrastructure and capacity needs survey, training feedback surveys, observations from cohort meetings and trainings, tracking attendance and milestone completion, and a summary of institutional activities and participant impacts and outcomes.

### Program Objectives and Outcomes

Expected outcomes were developed based on program objectives. Outcomes of the ALP are established from metrics recorded, achievement of program objectives, and participant feedback throughout the program.

**Table 1:** Summary of Objectives, Methods, and Outcomes

Objectives	Method(s)	Outcomes
1. Identify facilitators, barriers, and opportunities	Onboarding survey IT survey (participant) Evaluation discussion questions/guide; Technical Assistance Evaluation discussion questions/guide; Technical Assistance Mid-Contract Report Final Report	Increased understanding of the needs of biomedical and public health data needs of participating institutions
2. Provide training opportunities for library workers to build skills and capacity, related to the *All of Us* Program, within existing staff	Engagement activities (e.g., group meetings, Teams dashboard) Trainings/Technical Assistance (TA)/Resources	Built a network of librarians at institutions with a historic and current commitment to, or track record of, educating underrepresented students Expanded the number of institutions with a Data Use and Registration Agreement (DURA) in place Increased library worker access to Researcher Workbench Library workers' ability to articulate value of the Workbench to their campus communities Increased library workers' skill in basic coding to facilitate campus community Workbench usage
3. Position the library as a place for the campus community to gain *All of Us* data knowledge, skills, and abilities	Leadership buy-in resource template TA/Engagement and budget plan resources Final engagement plan	Increased infrastructure and capacity for use of the *All of Us* Researcher Workbench to conduct biomedical and public health data science research
4. Provide Guidance for library workers to create a resource to raise awareness of the *All of Us* Researcher Workbench and communicate availability of resources to the campus community	Short list of relevant *All of Us* resources, Data Support Hub, DRC Dissemination (developed publications/manuscripts) Conferences attended/presentations	Increased online presence and promotion of Researcher Workbench Increased awareness of the *All of Us* Data Training and Engagement for Academic Libraries Program
5. Provide a series of data engagement activities for library workers to build skills to provide data engagement	Research Workbench data engagement activities	Library workers hosted data engagement activities related to the Researcher Workbench

### Objective 1: Identify facilitators, barriers, and opportunities

The expected outcome of Objective 1 was to increase understanding of biomedical and public health data needs of participating institutions.

#### Pre-Program Onboarding and Evaluation Questions

Participants were asked about their expectations and concerns regarding participation. Eighty-two participants (71.3%) provided responses. Additionally, 25 institution representatives completed a Capacity and Infrastructure Survey to assess institutional access to data science tools, cloud computing capabilities, and IT policies. Results revealed participants’ expectations, technological landscape insights, and barriers.

#### Facilitators and Opportunities

Participants reported goals of providing data access tools and resources, raising awareness and usage of the Researcher Workbench, and increasing capacity and infrastructure for data science from program involvement. Other goals included engagement with their institution’s faculty and students, collaboration with other program participants, and enhanced knowledge of data science and analysis.

#### Barriers and Challenges

Main concerns about ALP participation included lack of administrative support, length of time to obtain the Data User Code of Conduct (DUCC), and staff capacity and availability.

Participants were asked to complete an end-of-program evaluation that included questions about barriers and challenges. Overall challenges faced were institutional barriers, funding limitations, and lack of pre-existing data science training programs. Common issues for accessing *All of Us* data were lack of dedicated computers and workstations required for secure access, data use knowledge gaps, training requirements to obtain access to the Researcher Workbench, VPN access, and institutional approval processes.

IT policies and access restrictions created another barrier. Disparities in available resources were highlighted by the number of institutions without cloud vendor agreements (52%) and institutions without data science workstations, high-performance computing capabilities, nor cloud vendor agreements (24%).

### Objective 2: Provide training opportunities for library workers to build skills and capacity

The expected outcomes of Objective 2 were to:

- Develop a network of 30–90 library workers.
- Expand the number of institutions with a Data Use and Registration Agreement (DURA) in place.
- Increase participant access to the Researcher Workbench by at least 60%.
- Increase the ability of library workers to articulate the value of the Researcher Workbench.
- Enhance understanding of the basics of using R and R Studio.

#### Library Program Statistics

Of the 115 library workers participating in the ALP, 67% reported obtaining their DUCC agreement, allowing access to the Researcher Workbench. The four institutions that did not have a DURA in place obtained one during the program.

#### Trainings and Cohort Meetings

Eight trainings were developed and offered 20 times over the course of the program. Twelve cohort meetings were also offered. Participants reported high percentages of satisfaction (indicating “strongly agree” or “agree”) for all trainings.

**Figure 1:**
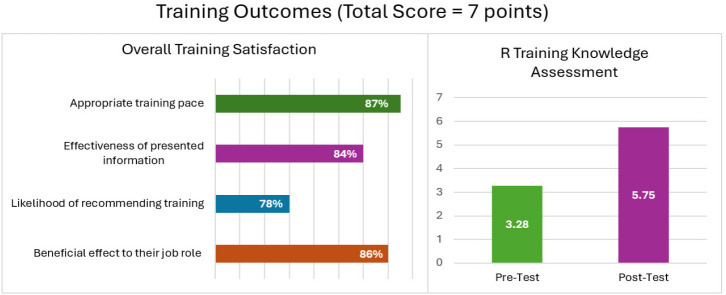
ALP Training Outcomes

Results to assess knowledge gained during the R for *All of Us* Basic Coding Training for Academic Libraries trainings included matching pre- and post-test responses from 40 learners and showed an increase in knowledge. Post-test scores were 2.47 points higher than pre-test scores (95% CI [-2.89, -2.06]) and found to be statistically significant (t (39) =-12.04, p<0.001). Please see Figure 1 for all training outcomes.

### Objective 3: Position the library as a place for the campus community to gain *All of Us* data knowledge, skills, and abilities

The expected outcome of Objective 3 was for participating institutions to have the infrastructure and capacity to conduct research using the Researcher Workbench. Institutions were provided with Capacity Building Awards funding support for key resources. Uses of the funding were broken into four categories as outlined in Table 2.

**Table 2:** Capacity Building Award Categories

Data Engagement Activity	General Outreach and Promotion	Enhancing Infrastructure	Staff/Personnel Time
**N=26**	**N=15**	**N=25**	**N=27**
**Examples**			
Engage computer science faculty to support training for programming in R or Python	Provide stipends for users to attend workshops on the Workbench and complete registration	Establish a dedicated space for Researcher Workbench access in the library or in a departmental building	Contract faculty to develop instructional module design
Collaborate with Ethical Learning Community	Creation of a fund for publication costs for researchers publishing using *All of Us* data	Laptop lending for take-home access	Hire student workers to assist with outreach and promotion
Workshops (e.g., coding training, collaboration)		High-performance servers	Attending workshops
Data scavenger hunts		Subscriptions to data lab instructions software	Developing projects using program data
Honors College Summer Cohort and curriculum creation			Develop a faculty learning community

### Objective 4: Develop a resource to raise awareness and communicate the availability of the Researcher Workbench

The expected outcome of Objective 4 was to increase the presence and promotion of the Researcher Workbench. At the completion of the program, 97% of institutions had developed online resource guides which were promoted via library websites, social media channels, newsletters, campus-wide presentations, and research-focused events. The creation of a library resource guide enhanced participants’ skills in resource development. See Appendix D for a list of resource guides created as part of the ALP.

### Objective 5: Provide a series of data engagement activities for library workers to build skills to provide data engagement

The expected outcome of Objective 5 was for library workers to host Researcher Workbench data engagement activities. The ALP modeled 11 data engagement activities that could be replicated on participant campuses. A total of 224 library workers attended these activities. Examples of engagement strategies planned by institutions can be seen in the Data Engagement Activity column of Table 2.

## DISCUSSION

The ALP advanced the research capacity and data engagement capabilities of academic libraries, particularly those serving underrepresented students. By raising awareness and increasing use of the *All of Us* Researcher Workbench [3], the ALP enhanced academic library workers’ skills in biomedical and public health data, as well as their library's research capacity. Enhancing the data science expertise of library workers empowered them to serve as an invaluable resource within their campus communities. Integrating the *All of Us* Researcher Workbench into library resources positioned libraries as a vital hub for faculty, researchers, and students seeking advanced tools for health-related research.

Participants reported highly favorable outcomes from participating in the ALP in terms of partnerships in data research, professional growth, and an increase in scholarly research contributions.

### Impact of the *All of Us* Academic Libraries Program

Participation in the ALP influenced individuals, libraries, and institutions by enhancing collaboration, professional development, research capacity, and institutional visibility. The program led to the expansion of data science knowledge, creation of new research resources, and strengthening of interdisciplinary partnerships.

### Professional Development and Skill Building

#### Collaboration and Networking

Participants reported an increase in collaborations with faculty, staff, and external institutions, resulting in joint research projects, faculty engagement, and institutional networking expected to continue beyond the program. One participant stated, “The connections I have been able to cultivate with my liaison area faculty have been far-reaching, and my interactions with those departments have never been busier.”

#### Expansion of Data Science and Research Skills

Many participants developed expertise in data science tools, improving their ability to support researchers and students. Topics such as data bias, data sovereignty, and precision medicine were frequently mentioned as valuable. One program administrator reported that “The Data Engagement Award and our Capacity Building Plan activities has positively impacted the library's perception on campus as a place where researchers can rely on our expertise and experience in working with secondary data.”

#### Expansion of Scholarly Research Contributions

Participants increased their scholarly accomplishments by authoring and collaborating on journal articles and posters or presenting at campus research events and professional conferences. See Appendix E for a list of participants’ scholarly contributions.

#### Career Growth and Leadership Opportunities

Career development opportunities were valuable for those new to health sciences and data librarianship. ALP trainings offered participants continuing education credits towards the Data Services Specialization (DSS) certification from the Medical Library Association (MLA). Participants used the experience to gain career advancement, leadership skills, training and outreach skill enhancements, and to pursue tenure. One participant shared, “This opportunity has been a great catalyst for me as I get started in the field of health sciences and data librarianship. Participating has really helped me hit the ground running in learning about my new role.”

#### Grant Writing and Funding Experience

Participants gained first-hand experience managing grants and navigating institutional processes for securing and utilizing funding, which will help them effectively navigate future grant applications.

### Library Impact and Research Support

#### Expansion of Library Resources and Services

Libraries expanded offerings with new research support services, data science training, and publication funding. The Researcher Workbench became a critical resource, increasing faculty and student engagement with biomedical and public health research. One library worker stated, “This award allowed me to develop services and programs that are already making a positive impact in our university community and beyond.”

#### Increased Institutional Visibility

The program raised the profile of and strengthened the position of participating libraries on their campus, even receiving recognition from university leadership. The library's role as a research hub has grown, with more faculty and students seeking library support for data-driven projects. One program administrator shared, “The program has had a transformative impact on the library by building a core group of faculty and staff with advanced data skills and knowledge. This has positioned the library as a critical partner in supporting data-driven research on campus. The increased engagement with the *All of Us* Researcher Workbench has led to new opportunities for collaboration between the library and various academic departments, further expanding the library’s role in research support and faculty development.”

### Institutional Impact and Research Growth

#### Interdisciplinary Collaboration

Libraries engaged with a diverse range of academic departments, including nursing, public policy, computer science, and agriculture. This interdisciplinary approach broadened research opportunities and fostered a culture of data literacy and health equity on campus.

#### Enhancing Research Capacity and Data Access

Institutions saw increased research activity using the *All of Us* dataset. Two reported transitioning from individual to institutional DURAs, reducing barriers to research and expanding access to the campus community.

#### Strategic Advancement in Research Classification

Participation in this program boosted research outputs, interdisciplinary engagement, and funding opportunities that benefit institutions pursuing Carnegie Classification of Institutions of Higher Education research designation (e.g., achieving R1 status). One participant reported, “[My institution] has aspirations of being a Carnegie R1 institution. I personally think that the level of interest generated in the *All of Us* program and the Researcher Workbench will play a pivotal role in enhancing the research outputs of this university and moving us toward that R1 goal.” Since participating in the ALP, five institutions pursued upgrading their research designation, with two institutions successfully attaining R1 status and three recognized as R2, at the time of publication.

### Limitations

While the program successfully contributed to enhancing institutional capacity and the skills of librarians, faculty, and students, there were limitations in the data collection process, impacting the ability to measure outcomes quantitatively. Although participants provided multiple instances of anecdotal evidence, the evaluation lacked standardized quantitative metrics, limiting the ability to report the full impact of the program. Despite these challenges, all program objectives were met, demonstrating the program's effectiveness in advancing research capacity and data engagement. Future evaluations should incorporate metrics from each participant institution including the number of workshops or events conducted, partnerships or collaborations established, and registered users before and after the program. Addressing these gaps in future evaluations could provide a more comprehensive assessment of the program's outcomes.

Although funding for the program was discontinued [5], its resources and strategies remain valuable tools for advancing equitable health research. Libraries and library workers can still utilize program resources to raise awareness of the Researcher Workbench’s existence and value found in a digital toolkit [6] created as part of an immersive presentation [7] at the MLA 2024 Annual Conference.

## CONCLUSION

The ALP set out to position academic libraries, already the center of campus communities, as a valuable resource for data-driven research. All program objectives were met, and the reported achievements and impacts from participants deemed the ALP a success, with an increase in library worker skills, which strengthened the position of the library to assist with data-driven research.

Though the ALP specifically used the Researcher Workbench data platform, the utilization of academic libraries as a central point for outreach and training could be replicated for future use with other data science program models.

## SUPPLEMENTAL FILES

**Appendix A:** ALP Participating Institutions

**Appendix B:** ALP Activity Milestones Chart

**Appendix C:** ALP Trainings and Descriptions

**Appendix D:** Participant Library Resource Guides

**Appendix E:** ALP Participant Scholarly Contributions
