# Supplementary material for: Advancing data-driven health research from the All of Us data training and engagement program
Source: J Med Libr Assoc. 2026 Jul 14;114(3):297–305. doi: 10.5195/jmla.2026.2324 (PMC13367302; doi:10.5195/jmla.2026.2324)
Supplement: Supplementary file 1 — Appendix A: ALP Participating Institutions [file jmla-114-3-297-s01.pdf]

Appendix A: ALP Participating Institutions

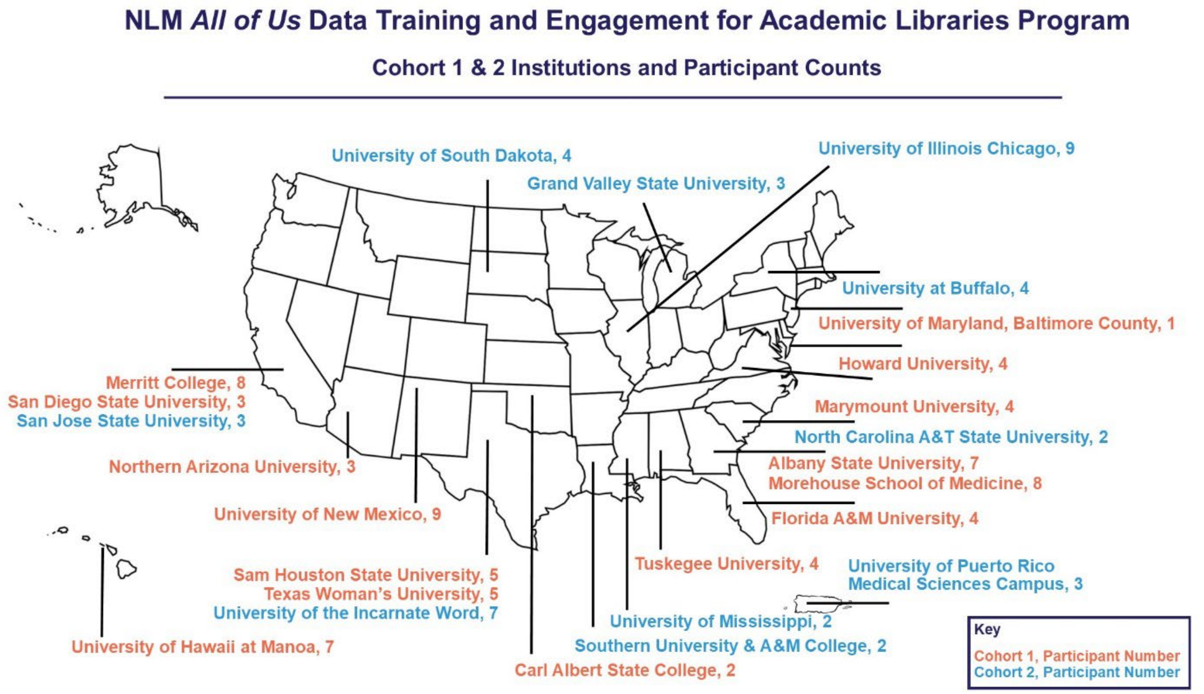

Pilot Cohort	State	Designation*
Albany State University	GA	HBCU
Carl Albert State College	OK	NASNTI
Florida A&M University	FL	HBCU
Howard University	D.C.	HBCU
Marymount University	VA	HSI
Merritt College	CA	HSI
Morehouse School of Medicine	GA	HBCU
Northern Arizona University	AZ	HSI
Sam Houston State University	TX	HSI
San Diego State University	CA	AANAPISI & HSI
Texas Woman's University	TX	HSI
Tuskegee University	AL	HBCU
University of Hawai'i at Manoa	HI	ANNHI
University of Maryland – Baltimore County	MD	AANAPISI
University of New Mexico	NM	HSI
**Cohort 2**	**State**	**Designation**
Grand Valley State University	MI	N/A
North Carolina Agricultural and Technical State University	NC	HBCU
San Jose State University	CA	AANAPISI & HSI
Southern University A&M College	LA	HBCU
University at Buffalo	NY	N/A
University of Illinois at Chicago	IL	AANAPISI & HSI
University of Mississippi	MS	N/A

University of Puerto Rico Medical Sciences Campus	PR	HSI
University of South Dakota	SD	N/A
University of the Incarnate Word	TX	HSI

*Minority Serving Institution (MSI) Designation [8]
