# Supplementary material for: Advancing data-driven health research from the All of Us data training and engagement program
Source: J Med Libr Assoc. 2026 Jul 14;114(3):297–305. doi: 10.5195/jmla.2026.2324 (PMC13367302; doi:10.5195/jmla.2026.2324)
Supplement: Supplementary file 2 — Appendix B: ALP Activity Milestones Chart [file jmla-114-3-297-s02.pdf]

Appendix B: ALP Activity Milestones Chart

BUILD AND STRENGTHEN THE RESEARCH CAPACITY OF ACADEMIC LIBRARIES											
**Activity 1**	Onboarding Meeting										
**Activity 2**	Participants sign up for *All of Us* Researcher Workbench, complete training, and sign data user code of conduct										
**Activity 3**		Collaborate to complete Capacity and Infrastructure Survey	Capacity and Infrastructure Survey Due								
**Activity 4**			Develop a Capacity Building Plan with Budget			Capacity Building Plan with Budget Due	Deploy Capacity Building Plan				
**Activity 5**		Develop and publish resource on your library website to raise awareness of the *All of Us* Researcher Workbench				Promote a resource on your library website to raise awareness of the *All of Us* Researcher Workbench					
**Activity 6**	Engage and Network with Other Cohort Participants										
**Activity 7**		Cohort Meeting	Cohort Meeting	Cohort Meeting	Cohort Meeting		Cohort Meeting		Cohort Meeting		Cohort Meeting
**Activity 8**	Training	Training	Training	Training	Training	Training		Training	Training		
**Activity 9**	Engage campus leadership, solicit feedback and buy-in										
**Activity 10**	Ongoing Evaluation				Mid-Contract / Agreement Report Due	Ongoing Evaluation					Final Report Due
	Ongoing Technical Assistance from Academic Libraries Program Staff										
