# Supplementary material for: Advancing data-driven health research from the All of Us data training and engagement program
Source: J Med Libr Assoc. 2026 Jul 14;114(3):297–305. doi: 10.5195/jmla.2026.2324 (PMC13367302; doi:10.5195/jmla.2026.2324)
Supplement: Supplementary file 3 — Appendix C: ALP Trainings and Descriptions [file jmla-114-3-297-s03.pdf]

Appendix C: ALP Trainings and Descriptions

Training	Description	Learning Objectives
An Overview of the *All of Us* Researcher Workbench and the Role of the Librarian<br><br>One Hour	This online training will feature the *All of Us* Data and Research Center (DRC) and will present more in-depth information on the Researcher Workbench and how campus libraries are well situated to respond to the needs of academic institutions for data-driven research; provide access to a diverse, longitudinal dataset; utilize training materials to assist campus communities; and introduce the *All of Us* database to researchers from various disciplines.<br><br>1 Continuing Education Credit from MLA Offered	• Enhance knowledge of the *All of Us* dataset and Researcher Workbench<br>• Increase understanding of the *All of Us* Researcher Workbench's value to your campus community<br>• Understand how to leverage your role as a librarian in campus-wide use of the *All of Us* Research Program<br>• Identify research fields that will benefit from access to the dataset
Introduction to Data Engagement Activities for Campus Communities<br><br>Two Hours	This two-hour, online training will walk through some unique and flexible engagement options for building community, solving problems, and applying the new skillsets needed to work in the Researcher Workbench and create interdisciplinary awareness and interest. We will provide an overview of hackathons, co-labs, challenges, sandboxes, and more, as well as discuss who participates and how they will benefit.<br><br>2 Continuing Education Credits from MLA Offered	• Understand some of the different types of engagement activities<br>• Learn, in brief, how to run different engagement activities<br>• Learn about the motivations of participants<br>• Learn about matching goals with activity type
Open Discussion of DURA Administration, the Individual Data User Code of Conduct Requirements, and the Relationship to Research Data Management	This online training will provide more details on the *All of Us* model of data engagement with members of the *All of Us* Access Team. A policy review will be provided by members of the policy team. Various scenarios that library workers might encounter will be presented along with a discussion on what their role and expectations will be in various scenarios.	• Understand the *All of Us* Model of Data Access<br>• Understand the policies that come with utilizing the *All of Us* Researcher Workbench<br>• Apply knowledge learned to possible situations

One Hour	1 Continuing Education Credit from MLA Offered	
Tools and Ideas for Promoting the Researcher Workbench on Your Campus<br><br>One Hour	Intended to assist in raising awareness of the Researcher Workbench amongst campus communities, this online training will provide strategies to help with disseminating information and developing tools to reach both emerging and established researchers across multiple fields of study.<br><br>1 Continuing Education Credit from MLA Offered	• Identify strategies for creating and tailoring your marketing message<br>• Develop a toolkit of methods and locations to promote your message<br>• Gather tools for promoting the Workbench to both emerging and established researchers of various disciplines
Data Scavenger Hunt<br><br>One and a Half Hours	Using a scavenger hunt model [9], groups of participants will interactively engage with and explore in the data browser, which will provide an interactive overview of the data types and potential questions that can be surfaced using *All of Us* datasets.	• Learn to navigate the landing page at: https://www.researchallofus.org/ including data snapshots, data sources, and research progress<br>• Learn more about the types of data found in the browser<br>• Find specific data in the browser<br>• Identify new information for potential follow-up explorations
Navigating the Researcher Workbench<br><br>One Hour	This online training will be a full walkthrough of the Researcher Workbench. Learn about the various components and how to ensure you aren't accruing computational costs accidentally. Gain confidence in showing the Workbench to others.	• Learn to navigate the Research Workbench<br>• Learn to create, find, and close analysis environments<br>• Learn to share workspaces
Using Point and Click Tools in the Researcher Workbench<br><br>One Hour	This online training will feature the *All of Us* Data and Research Center (DRC) providing live demonstrations of the Cohort Builder and Dataset Builder tools within the Researcher Workbench.	• Learn about the different data available within the *All of Us* Research Program and how they are organized<br>• Observe how to start a project<br>• Explore Cohort Builder and Dataset Builder
Basic Coding Training Series<br><br>10 Hours	Dive into R and discover its endless possibilities, including unlocking the power of data analysis and visualization and the coding skills	• Have a basic understanding of how to create and duplicate workspaces in the *All of Us* Researcher Workbench

	required to fully utilize the *All of Us* Researcher Workbench datasets.<br><br>The two-day, live instruction online course will cover installing/loading packages, loading data, creating data objects, learning basic R commands used to explore data, shaping and filtering data, writing data/graphics into output files, visualizing, and merging data.<br><br>This is an introductory course on the R programming language designed to cater to individuals of all backgrounds. No prerequisites or coding experience are required. Confirmation of access to the Researcher Workbench must be on file with *All of Us* Data Training and Engagement for Academic Libraries Program staff prior to registration.<br><br>4 Continuing Education Credits from MLA Offered	• Understand how to use the interactive *All of Us* Researcher Workbench tools to build cohorts and datasets<br>• Can identify the fundamental data structures for analysis and visualization<br>• Have a basic understanding of coding in the R language<br>• Have a basic understanding of how to combine different data types<br>• Can perform common data manipulation and visualization tasks using R Studio in the *All of Us* Researcher Workbench
