# Supplementary material for: Advancing data-driven health research from the All of Us data training and engagement program
Source: J Med Libr Assoc. 2026 Jul 14;114(3):297–305. doi: 10.5195/jmla.2026.2324 (PMC13367302; doi:10.5195/jmla.2026.2324)
Supplement: Supplementary file 4 — Appendix D: Participant Library Resource Guides [file jmla-114-3-297-s04.pdf]

Appendix D: Participant Library Resource Guides

* Denotes Cohort 1 Institution

^ Denotes Cohort 2 Institution

Institution	Web Address	Views	Time Frame
* Albany State University	https://libguides.asurams.edu/aouresearcherworkbench	305	September 2023 – March 2025
* Carl Albert State College	https://carlalbert.edu/academics/library/	Data was not reported at the time of publication.	
^ Grand Valley State University	https://libguides.gvsu.edu/allofus	581	April 2024 – March 2025
* Howard University	https://hsl.howard.edu/allofus	154	March 2024 – March 2025
* Marymount University	https://marymount.libguides.com/AOUResearchWorkbench	386	August 2023 – March 2025
* Merritt College	https://merritt.libguides.com/c.php?g=1349153	Data was not reported at the time of publication.	
* Northern Arizona University	https://libraryguides.nau.edu/allofus	875	August 2023 – March 2025
^ North Carolina Agricultural and Technical State University	https://libguides.library.ncat.edu/allofus	Data was not reported at the time of publication.	
* Sam Houston State University	https://shsulibraryguides.org/AoU	1647	June 2023 – March 2025
* San Diego State University	https://libguides.sdsu.edu/allofus	358	November 2023 – March 2025
^ San Jose State University	https://libguides.sjsu.edu/allofus	981	March – September 2024
^ Southern University Agricultural and Mechanical College	https://subr.libguides.com/ALLOFUS	439	February 2024 – October 2024
* Texas Woman's University	https://libguides.twu.edu/allofus	401	September 2023 – November 2024
* Tuskegee University	https://tuskegee.libguides.com/c.php?g=1350060	113	June 2024 – March 2025
^ University at Buffalo	https://research.lib.buffalo.edu/allofus	810	March – September 2024
* University of Hawai'i at Manoa	https://guides.library.manoa.hawaii.edu/All-of-Us/what-is-all-of-us<br>https://hslib.jabsom.hawaii.edu/all-of-us	Data was not reported at the time of publication.	
^ University of Illinois Chicago	https://researchguides.uic.edu/allofus	509	April–September 2024

^ University of the Incarnate Word	https://libguides.uiwtx.edu/allofus/home	225	March – September 2024
* University of Maryland, Baltimore County	https://lib.guides.umbc.edu/c.php?g=1337902&p=9859749&preview=734db44cd0c1e8fcb7f315498cddc922	350	August 2023 – March 2025
^ University of Mississippi	https://guides.lib.olemiss.edu/allofus	1118	March – December 2024
* University of New Mexico / University of New Mexico Health Sciences Center	https://libguides.unm.edu/allofus https://libguides.health.unm.edu/allofus	1708	November 2023 - March 2025
^ University of Puerto Rico Medical Sciences Campus	https://rcm-upr.libguides.com/c.php?g=1390933	597	March – September 2024
^ University of South Dakota	https://libguides.usd.edu/AllOfUsResearchProgram	297	March 2024 – February 2025
