# Supplementary material for: Advancing data-driven health research from the All of Us data training and engagement program
Source: J Med Libr Assoc. 2026 Jul 14;114(3):297–305. doi: 10.5195/jmla.2026.2324 (PMC13367302; doi:10.5195/jmla.2026.2324)
Supplement: Supplementary file 5 — Appendix E: ALP Participant Scholarly Contributions [file jmla-114-3-297-s05.pdf]

Appendix E: ALP Participant Scholarly Contributions

This list of scholarly works reflects the contributions self-reported by participants and may not represent the full scope of achievements across the program.

Published Abstracts

- Flores Rivera E, Seguí Caballero CW, Roche Lima A, Meléndez Berríos JS, Ubieta Santiago JR, Ramos Llera D, Del Valle López PA, Barrios Llorens R, Meléndez Ayala T, Cruz Zayas A, Santiago Hernández M. Advancing health research through library-led training: The *All of Us* Researcher Workbench Initiative at the University of Puerto Rico Medical Sciences Campus [abstract]. P R Health Sci J. 2025 Apr;44(1 Suppl):38. Available from: https://prhsj.rcm.upr.edu/index.php/prhsj/issue/view/157
- Maldonado A, Cintrón M, Lorenzo N, Melin K, Hernández J, Rivera S, Torres B. Determining the association of antiretroviral treatment, sociodemographic factors, social determinants of health, and depression in patients with HIV [abstract]. P R Health Sci J. 2025 Apr;44(1 Suppl):201. Available from: https://prhsj.rcm.upr.edu/index.php/prhsj/issue/view/157
- Santiago MG, Haedo-Cruz MS, Vélez Crespo G, Borges-Vélez G, Pérez-Santiago J. A cohort analysis using NIH *All of Us*: Cancer diagnostic age and incidence in ADHD versus non-ADHD population [abstract]. P R Health Sci J. 2025 Apr;44(1 Suppl):186. Available from: https://prhsj.rcm.upr.edu/index.php/prhsj/issue/view/157

Presentations/Posters

- He, Y. Engineering librarian on the table: Facilitating interdisciplinary research using the *All of Us* datasets on campus [Lightning Talk]. Medical Library Association (MLA) Annual Conference, Pittsburgh, PA. (2024, June 23-26).
- He, Y., Szydlowski, N., Hackman, D., & McNiece, Z. (2025, April 29 – May 2). Implementing the NLM *All of Us* Academic Libraries Program at a minority-serving institution pursuing an R2 designation [Program presentation].
- Hughes, B., Shumaker, J., Stokan, E., & Yohannes, S. (2024, September). Resource sharing and engagement: a cross-campus approach to enhancing student and researcher access to biomedical data. Provost's Teaching & Learning Symposium, Baltimore, Maryland. Available from: https://calt.umbc.edu/programs/provosts-teaching-and-learning-symposium/
- Liu, G., He, Y., Huang, G., & Luo, Y. Engineering Librarian on the Table: Facilitating Interdisciplinary Research Using the *All of Us* Datasets on Campus. American Society for Engineering Education (ASEE) – Engineering Libraries Division (ELD) Annual Conference & Exposition, Portland, OR. (2025, March 30 – April 2). Available from: https://osf.io/45m8y/
- Liu, G., He, Y., Huang, G., & Luo, Y. Leveraging machine learning algorithms to predict self-rated physical health: Insights from the *All of Us* dataset [Poster Presentation]. International Symposium on Human Factors and Ergonomics in Health Care, Toronto, Canada.
- Rethlefsen ML, Rhue D. *All of Us* at University of New Mexico. In Division of Engagement and Outreach at *All of Us* meeting, August 27, 2024. Virtual; 2024 [invited lightning talk].

- Rhue DJ, Quinn T, Sloane L, and Benedict, K. *All of Us*: Using Big Data to Study Factors Affecting Health and Health Impacts. University of New Mexico, Tech Days, April 26, 2024. [invited conference presentation]. Available from: https://digitalrepository.unm.edu/hslic-posters-presentations/147/
- Rhue DJ and Sloane L. The *All of Us* Research Program at the University of New Mexico. In Love Data Week, February 10, 2025. Virtual [invited presentation to international audience].
- He, Y., Szydlowski, N., Hackman, D., & McNiece, Z. (2025, April 29 – May 2). Implementing the NLM *All of Us* Academic Libraries Program at a minority-serving institution pursuing an R2 designation [Program presentation].
- He, Y. Engineering librarian on the table: Facilitating interdisciplinary research using the *All of Us* datasets on campus [Lightning Talk]. Medical Library Association (MLA) Annual Conference, Pittsburgh, PA. (2024, June 23-26).
